# Duplicated Hox genes in the spider *Cupiennius salei*

**DOI:** 10.1186/1742-9994-4-10

**Published:** 2007-03-13

**Authors:** Evelyn E Schwager, Michael Schoppmeier, Matthias Pechmann, Wim GM Damen

**Affiliations:** 1 Institute for Genetics, University of Cologne, Zülpicher Straße 47, 50674 Köln, Germany; 2 Friedrich-Alexander University Erlangen, Institute for Biology, Department of Developmental Biology, Staudtstr. 5, D-91058 Erlangen, Germany

## Abstract

**Background:**

Hox genes are expressed in specific domains along the anterior posterior body axis and define the regional identity. In most animals these genes are organized in a single cluster in the genome and the order of the genes in the cluster is correlated with the anterior to posterior expression of the genes in the embryo. The conserved order of the various Hox gene orthologs in the cluster among most bilaterians implies that such a Hox cluster was present in their last common ancestor. Vertebrates are the only metazoans so far that have been shown to contain duplicated Hox clusters, while all other bilaterians seem to possess only a single cluster.

**Results:**

We here show that at least three Hox genes of the spider *Cupiennius salei *are present as two copies in this spider. In addition to the previously described duplicated *Ultrabithorax *gene, we here present sequence and expression data of a second *Deformed *gene, and of two *Sex comb reduced *genes. In addition, we describe the sequence and expression of the *Cupiennius proboscipedia *gene. The spider *Cupiennius salei *is the first chelicerate for which orthologs of all ten classes of arthropod Hox genes have been described. The posterior expression boundary of all anterior Hox genes is at the tagma border of the prosoma and opisthosoma, while the posterior boundary of the posterior Hox genes is at the posterior end of the embryo.

**Conclusion:**

The presence of at least three duplicated Hox genes points to a major duplication event in the lineage to this spider, perhaps even of the complete Hox cluster as has taken place in the lineage to the vertebrates. The combined data of all *Cupiennius *Hox genes reveal the existence of two distinct posterior expression boundaries that correspond to morphological tagmata boundaries.

## Background

Hox genes are found in all metazoan phyla. They are active in distinct domains along the main body axis and direct the morphogenesis of segment-specific structures via the activation of downstream target genes. Hox genes are important factors in the evolution of animal body plans. They share three key traits [1]: (1) they are basically organized in a cluster in the genome, (2) there is a correlation between the 3'-5' order of the genes in the genome and the anterior to posterior order of expression of the genes, and (3) the protein encoded by each of the genes contains a homeobox, a highly conserved 60 amino acid sequence that is a DNA binding motif [2].

The Hox genes primarily are involved in providing the embryo with positional information. This is most obvious from experiments with mutants that lack a particular Hox gene or from embryos in which a particular Hox gene is misexpressed. Such embryos produce structures at the incorrect position, as the affected cells seem to misunderstand their location within the embryo. For instance when a particular Hox gene is absent or is misexpressed in the fruit fly *Drosophila melanogaster*, the affected segments get the identity of another segment [e.g. [3,4]]. Famous examples are the four-winged *Drosophila *fly, in which the halteres on the third thoracic segments are transformed to wings, or the flies with legs at the position of the antennae. This is homeosis, and the mutations are homeotic transformations. The Hox genes thus act as selector genes that select one anterior-posterior identity over another along the main body axis in the embryo, while their downstream target genes actually act as realizator genes that make the structure specific for each location [summarized in [5,6]].

Due to the widespread sampling of Hox genes from a large variety of metazoans, the evolution of Hox genes is well characterized. Gene duplications played an important role in the evolution of the Hox genes. Recent data on cnidarians [7] suggest that the last common ancestor of the cnidarians and bilaterians had a Hox cluster consisting of two anterior genes (a *Hox1/2 *and a *Hox3 *gene), and that the Hox cluster subsequently expanded via internal duplications in the lineages leading to the cnidarians and the bilaterians. The last common ancestor of the bilaterians (animals with a bilateral symmetry) presumably still contained such a cluster of three genes as seen in today's acoel flatworms, which may represent the closest approximation of the ancestral bilaterian [8]. The last common ancestor of the other bilaterians (the protosome/deuterostome ancestor) at least contained seven different Hox genes, maybe even nine or more [9], implying several Hox gene duplication events in this lineage after the divergence of the acoel flatworms [8]. The different genes in the Hox complex are most likely the result of tandem duplications followed by sequence divergence [9,10]. In vertebrates the complete Hox cluster has been duplicated twice, presumably via whole genome duplications, resulting in four clusters in tetrapods, while in teleost fish additional duplication events took place [11].

Arthropod Hox genes can basically be assigned to ten different classes and seem to be present in a single Hox cluster [12]. In the chelicerates (spiders, scorpions, mites, ticks, horseshoe crabs) however there are examples of duplicated Hox genes. In a PCR survey Cartwright et al. [13] found 28 different small homeobox fragments of Hox genes in the horseshoe crab *Limulus polyphemus*. They could identify one to four representatives for each Hox gene class suggesting the presence of multiple Hox clusters in an invertebrate. Additional data for duplications of Hox genes come from two spiders, *Achaearanea tepidariorum *and *Cupiennius salei*. Two copies of the *Deformed *(*Dfd*) gene have been described for *Achaearanea *[14], and a duplicated *Ultrabithorax *(*Ubx*) gene has been described for *Cupiennius *[15]. In addition to these chelicerates there is one example of a duplicated Hox gene in a myriapod; a duplicated *Dfd *gene has been described for the geophilomorph centipede *Pachymerium ferrugineum *[16].

In the present paper we describe four new Hox genes from the spider *Cupiennius salei*: a *proboscipedia *gene (*Cs-pb*), a second *Dfd *gene (*Cs-Dfd-2*), and two *Sex comb reduced *(*Cs-Scr*) genes. Our data shows that at least three Hox genes (*Dfd*, *Scr*, and *Ubx*) are duplicated in the spider *C. salei*. Furthermore, *pb *and *Scr *orthologs have not been described from *C. salei *before. In previous work we described the sequence and expression of orthologs of eight classes of Hox genes from the spider *C. salei*: *labial *(*Cs-lab*), *Hox3 *(*Cs-Hox3*), *Deformed *(*Cs-Dfd-1*), *fushi tarazu *(*ftz*), *Antennapedia *(*Cs-Antp*), *Ultrabithorax *(*Cs-Ubx-1 *and *Cs-Ubx-2*), *abdominal-A *(*Cs-abdA*), and *Abdominal-B *(*Cs-AbdB*) [15,17-19]. With our new data on *pb *and *Scr*, the Central American wander spider *Cupiennius salei *becomes the first chelicerate for which orthologs of all ten arthropod Hox genes have been described.

## Results

### Spider Hox genes

**Figure 1:**
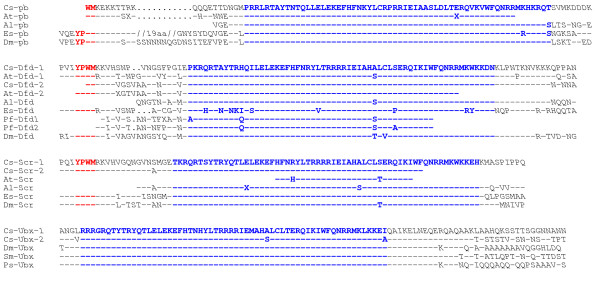
Alignment of chelicerate Proboscipedia, Deformed, Sex comb reduced, Ultrabithorax sequences. Aligned is a fragment covering the hexapeptide (core in red) and homeodomain (blue) plus some flanking sequences. The deduced proteins sequences are from the chelicerates *Cupiennius salei *(*Cs*; American wandering spider), *Achaearanea tepidariorum *(*At*; common house spider), *Archegozetes longisetosus *(*Al*; mite), *Endeis spinosa *(*Es*; seaspider) the fruit fly *Drosophila melanogaster *(*Dm*; insect), the centipedes *Pachymerium ferrugineum *(*Pf*; myriapod), *Strigamia maritima *(*Sm*; myriapod), and the isopod *Porcellio scaber *(*Ps*; crustacean). Amino acids identical to the upper sequence are indicated as dashed "-"; dots "." are used to indicate gaps that have been introduced for alignment purposes. The linker between hexapeptide and homeodomain in the *Es*-pb sequence is rather long, therefore 19aa are not shown (marked as //19aa// in the alignment). Please note, some of the *At, Al *and *Pf *sequences from the GenBank still contain ambiguous sites that result in an unknown amino acid in the deduced protein sequence; these are indicated as "X" in the alignment. Accession numbers of the sequences used: *Cs*: AM419029, CAA07498, AM419032, AM419030, AM419031, CAA07500, CAA07501; *At*: AF151997, AF151995, AF151996, AF151998; *Al*: AF071406, AF071403, AF071407; *Es*: ABD46724. ABD46727, ABD46728; *Dm*: CAA45272, NP_477201, NP_524248, NP_536752; *Pf*: CAB75743, CAB75746; *Sm*: ABD16212, *Ps*: BAE97002.

We isolated fragments of *proboscipedia *(*Cs-pb*), two copies of *Sex comb reduced *(*Cs-Scr-1 *and *Cs-Scr-2*), and a second copy of *Deformed *(*Cs-Dfd-2*) from the spider *Cupiennius salei*. Alignment with chelicerate and other arthropod sequences unambiguously show that these are *Cupiennius *orthologs of these Hox genes (Fig. 1). *pb *and *Scr *class Hox genes have not been recovered in *Cupiennius *before, but some data are available from other chelicerates: the mite *Archegozetes longisetosus*, the common house spider *Achaearanea tepidariorum *and the seaspider *Endeis spinosa *[14,19,21]. However, with the isolation of *pb *and *Scr *from *Cupiennius*, this spider is the first chelicerate species for which orthologs of all ten arthropod Hox gene classes are described.

More importantly, it becomes clear that at least three Hox genes in *Cupiennius *are present as duplicate copies. There are two *Dfd *orthologs [[15], this paper], two *Scr *orthologs (this paper), and two *Ubx *orthologs [15]. Despite the similarities in the amino acid sequence and especially within the homeodomain, the two copies are significantly different from each other on the DNA sequence level (not shown) and thus are different genes and not different alleles. At the moment it is unclear whether there are additional copies of other *Cupiennius *Hox genes. Abzhanov et al [14] also described two *Dfd *genes for another spider (*Achaearanea tepidariorum*), however, for one of them they only obtained a small PCR fragment encoding 27 amino acids within the highly conserved homeodomain. We recovered additional sequence information of this *Achaearanea Dfd *gene (*At-Dfd-1*) via RACE-PCR (Fig 1). The *Cs-Dfd-1 *[15] and *At-Dfd-1 *sequences are more similar to each other than to the newly identified *Cs-Dfd-2 *sequence or to the *At-Dfd-2 *[14] sequence. Also *Cs-Dfd-2 *and *At-Dfd-2 *are more similar to each other than to *Cs-Dfd-1 *or *At-Dfd-1*. This is most obvious from the sequence between hexapeptide and homeodomain. Based on the sequences (Fig. 1) we propose that *Cs-Dfd-1 *and *At-Dfd-1 *are gene orthologs and that *Cs-Dfd-2 *and *At-Dfd-2 *are gene orthologs. Also the expression patterns of *At-Dfd-1 *[14] and *Cs-Dfd-1 *[15] in the legs are remarkably similar (see also below). Therefore, the duplication of *Dfd *presumably was already present in the last common ancestor of these two spiders.

There is another arthropod, the geophilomorph centipede *Pachymerium ferrugineum *(Myriapoda), that contains two copies of the *Dfd *gene [16]. However, these two centipede *Dfd *genes are more similar to each other than to any of the spider genes (Fig. 1). In addition, no Hox gene duplications have been described for other myriapods, *e.g*. the centipede *Lithobius atkinsoni *[22] and the millipede *Glomeris marginata *[23]. This suggests that the two *Dfd *genes in *Pachymerium *are the result of an independent duplication event in the geophilomorph centipedes.

### Expression patterns of *Cs-pb*, *Cs-Dfd *and *Cs-Scr*

**Figure 2:**
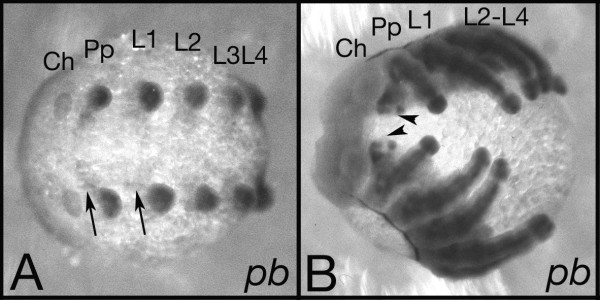
Expression pattern of the *Cs-pb *gene. (A) Limb bud stage, *proboscipedia *(*pb*) is expressed in the pedipalpal segment (Pp) and walking leg segments (L1-L4). The expression is most prominent in the appendages itself, but there is also some weaker expression in the ventral ectoderm (arrows). There is no expression in the cheliceral segment (Ch) or in the opisthosomal segments. (B) Inversion stage, *pb *is expressed in the appendages of the Pp and L1-L4 segments and in spots (arrowheads) in the ectoderm ventral to the appendages. Also at these older stages the cheliceral and opisthosomal segments are free of expression. Both embryos: ventral view.

The expression of the Hox genes was studied via in situ hybridizations. *Cs-pb *is expressed in the pedipalpal segment and the four walking leg segments (L1-L4) (Fig. 2). This is similar to the common house spider *Achaearanea *and the mite *Archegozetes longisetosus *[14,20]. *Cs-pb *thus is expressed in the same segments as *Cs-lab *and *Cs-Hox3 *[15,17] (see also summary in Figure 6). At the limb bud stage (Fig. 2A) the expression is most obvious in the appendages (pedipalps and walking legs) but there is also some expression in the ventral ectoderm. At the inversion stage (Fig. 2B) *Cs-pb *expression is also clearly visible in the ventral ectoderm. We never observed any expression in the cheliceral segment or the opisthosomal segments.

**Figure 3:**
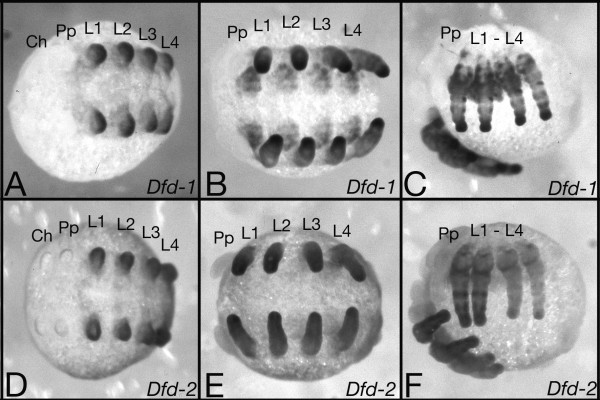
Expression pattern of the *Cs-Dfd-1 *and *Cs-Dfd-2 *genes. The *Cs-Dfd-1 *gene (A-C) and the *Cs-Dfd-2 *gene (D-F) are expressed in the four segments that bear the walking legs (L1-L4). Expression is seen in the appendages as well as in the ventral ectoderm. The ventral ectoderm expression of *Cs-Dfd-1 *is stronger than the one of *Cs-Dfd-2*, especially at the older stages (B and C compared with E and F). The expression patterns in the legs are different for the two genes (most obvious in C an F), while in addition, Cs-Dfd-2 expression in L3 and L4 is weaker compared to the expression in L1 and L2. The level of *Cs-Dfd-1 *expression is similar for all four legs. A and D: ventral view of limb bud stage, B and E: ventral view of limb stage before inversion, C and F: lateral view of inversion stage. There is never ever expression of any of the two spider *Dfd *genes in the cheliceral (Ch), pedipalpal (Pp), or opisthosomal segments.

Expression of the *Cs-Dfd-2 *gene is limited to the four segments that bear the walking legs (L1-L4) (Fig. 3D–F). These are the same segments that express the *Cs-Dfd-1 *gene (Fig. 3A–C). However, there are differences in the intrasegmental domains of the expression of the two *Cs-Dfd *paralogs. Neither are homogenously expressed, but each gene is expressed in a distinct pattern within the leg segments. Most prominent is the very strong expression of *Cs-Dfd-1 *at the most distal tip of the legs. Although *Cs-Dfd-2 *also is expressed in the distal tip, this expression is not as prominent as the one of *Cs-Dfd-1*. Furthermore, while *Cs-Dfd-1 *is expressed in all four walking legs at the same intensity (Fig. 3C), expression of *Cs-Dfd-2 *is weaker in L3 and L4 compared to L1 and L2 (Fig. 3F). Another difference is the strength of expression in the ectoderm ventral to the legs: *Cs-Dfd-2 *is only weakly expressed, while *Cs-Dfd-1 *is strongly expressed here (compare Fig 3A and Fig 3D). The common house spider *Achaearanea tepidariorum *also contains two *Dfd *genes [14]. Comparison of the expression pattern of the two *Cupiennius Dfd *genes with the two *Achaearanea Dfd *genes [14] shows that *Cs-Dfd-1 *and *At-Dfd-1 *show similarities in their expression patterns. Most typical is the strong expression at the distal tip of the leg, which is much less prominent for *Cs-Dfd-2 *and *At-Dfd-2*. This prominent expression in the tip of the leg of *Cs-Dfd-1 *is most obvious when the colour reaction of the in situ develops (not shown).

**Figure 4:**
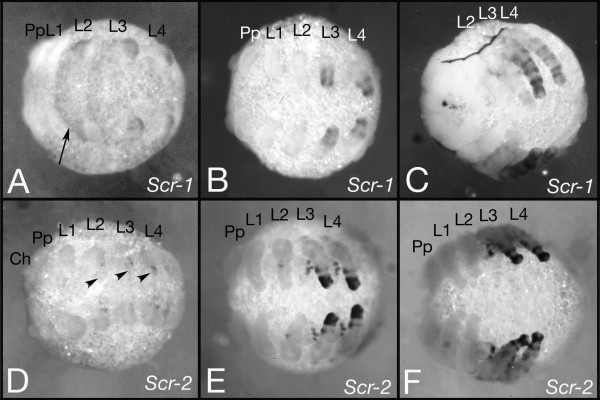
Expression pattern of the *Cs-Scr-1 *and *Cs-Scr-2 *genes. Expression of the *Cs-Scr-1 *gene (A-C) and the *Cs-Scr-2 *gene (D-F). At the limb bud stage *Scr-1 *is more or less homogenously expressed in L2, L3 and L4 (arrow in A). At later stages there is expression in rings in the legs of L3 and L4 (B) and later also weak expression in the legs of L2 (C). *Scr-2 *expression starts as small spots in the ventral ectoderm of L2, L3 and L4 (arrowheads in D) that persist during further development (E,F). Later there is also *Scr-2 *expression in the legs of L3 and L4, but in contrast to *Scr1 *we never observed expression in the legs of L2 (F). Ch: cheliceres, Pp: pedipalps, L1-L4: walking leg 1–4.

*Cs-Scr-1 *and *Cs-Scr-*2 also are expressed in similar but not identical patterns. Cs *-Scr-1 *is initially expressed in the second, third and fourth walking leg segment (L2-L4) (Fig 4A). In the appendages, expression first appears only in the walking legs of L3 and L4 (Fig. 4B) and only later, but weaker, in the walking legs of L2 (Fig 4C). *Cs-Scr-2 *is also initially expressed in L2-L4, but the expression is not as widespread as the *Cs-Scr-1 *expression is as it is restricted to some small spots in the ventral ectoderm (Fig. 4D). Later expression is seen in the legs of L3 and L4 (Fig. 4E) but we never observed expression of *Cs-Scr-2 *in the legs of L2 (Fig. 4F). As with the two *Dfd *genes the patterns within the legs also differ for the two *Cupiennius Scr *genes (compare Fig. 4C and Fig. 4F).

**Figure 5:**
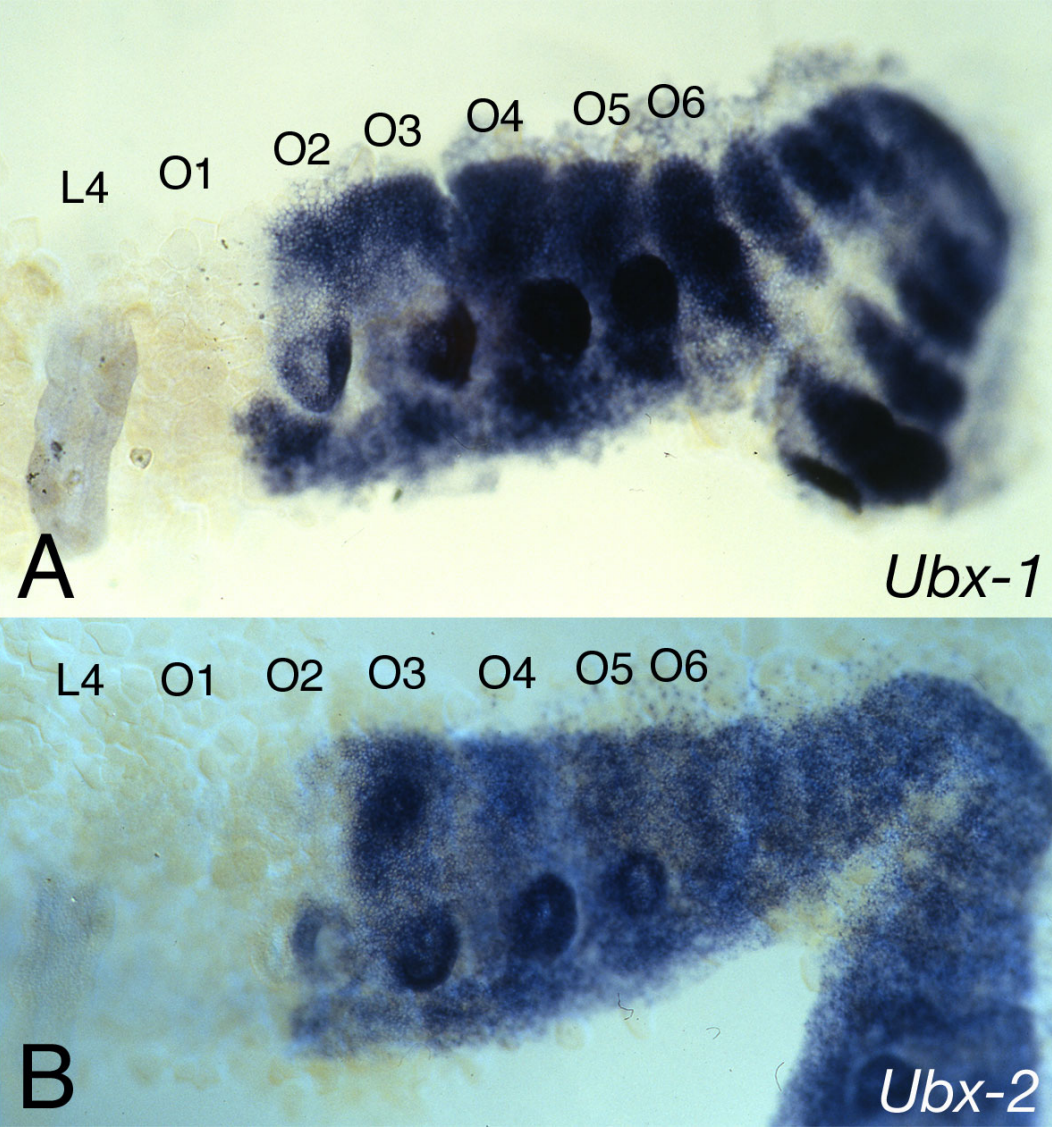
Expression pattern of the *Cs-Ubx-1 *and *Cs-Ubx-2 *genes. The anterior expression border of both *Ubx *genes is in the second opisthosomal segment (O2). The one of *Cs-Ubx-1 *(A) is at the anterior edge of O2, while the one of *Cs-Ubx-2 *is at posterior portion of O2 and corresponds to the parasegment boundary [15]. The opisthosomal limb primordia that will form the respiratory organs and spinnerets are visible on O2-O5. Abbreviations: L4: walking leg 4, O1-O6: opisthosomal segment 1–6.

The expression patterns for *Cs-Ubx-1 *and *Cs-Ubx-2 *have been described before [15]. The anterior border of *Cs-Ubx-1 *is slightly more anterior than that of *Cs-Ubx-2 *[15]. There are small intrasegmental expression differences between the two *Ubx *genes. *Cs-Ubx-2 *is more homogeneously expressed compared to *Cs-Ubx-1 *(Fig 5A,B).

**Figure 6:**
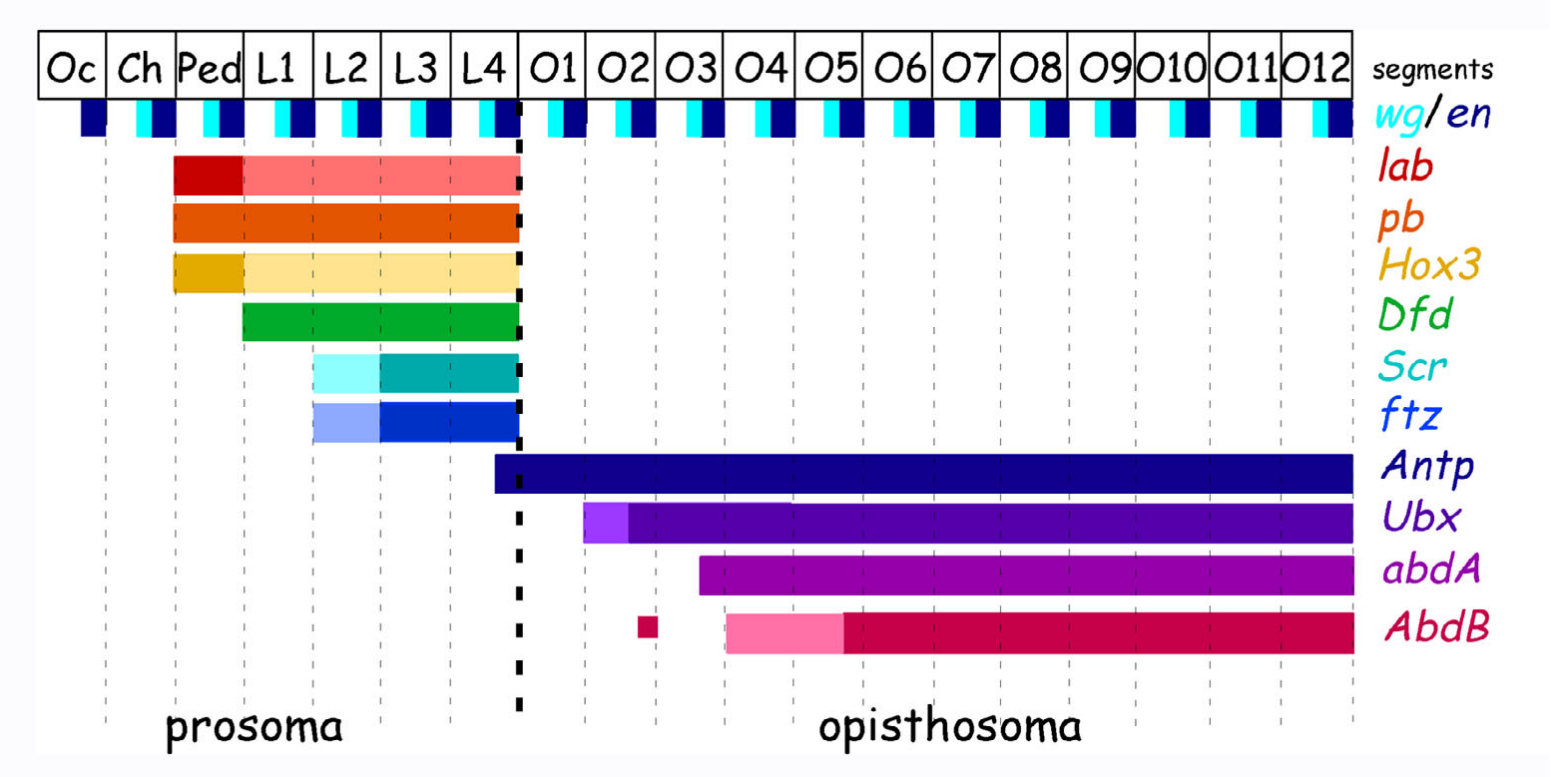
Schematic representation of the segmental expression patterns of the Hox genes in the spider *Cupiennius salei*. The segmental organization of the spider and the expression domains of *engrailed *(*en*; dark blue) and *wingless *(*wg*; bright blue) are shown at the top [see Ref. 31], below are the expression domains of the various Hox genes. Data from: this paper, Refs. [15, 17-19]. Note that the posterior expression domain of the anterior Hox genes (*lab, pb, Hox3, Dfd, Scr, ftz*) is in the segment of the fourth walking leg and corresponds to the tagma boundary between prosoma and opisthosoma. The expression of the posterior Hox genes (*Antp, Ubx, abdA, AbdB*) expands to the very posterior end of the embryo. Abbreviations: Oc: ocular; Ch: cheliceral segment; Pp: pedipalpal segment; L1-L4: walking leg segment 1–4; O1-O12: opisthosomal segment 1–12.

## Discussion

### All ten arthropod Hox genes are present in the spider *Cupiennius salei*

The combined data from other arthropods [e.g. [8,19,20]], summarized in Hughes and Kaufman [12], imply that the Hox complex of the last common ancestor of all arthropods contained ten Hox genes. The present data of the *Cs-pb *and *Cs-Scr *genes combined with our previous work [15,17-19] show that at least one copy of each of the ten arthropod Hox genes is present in the spider *Cupiennius *(Fig. 6). At least three of the Hox genes are even present as two copies (see below). The expression data on *Cs-pb *and *Cs-Scr *make *Cupiennius *the first chelicerate for which expression data are known for all ten different arthropod Hox genes; previously the chelicerate data were an assemblage from three different species [see also Ref. [12]].

### At least three duplicated Hox genes in the spider *Cupiennius salei*

Our data show that at least three Hox genes are present as two copies in *Cupiennius *[combined data from this paper and Ref. [15]]. There are previous reports on duplication of Hox genes in chelicerates. Cartwright et al. [13] could identify one to four representatives per Hox gene class in the horseshoe crab *Limulus polyphemus*. However, there is no expression data for the *Limulus *Hox genes. In the spiders *Achaearanea *[14] and *Cupiennius *[15], previous one duplicated Hox gene each had been described. For mite and pycnogonids no duplicated Hox genes have been described [e.g. [20,21]].

In all three cases in *Cupiennius *(*Dfd*, *Scr*, and *Ubx*), the two paralogs are expressed in comparable but not identical domains. They are expressed in the same segments with differences in the intrasegmental patterns. This shows a striking similarity with what is seen for the duplicated Hox genes of the various paralogous groups in vertebrates that are also expressed in similar but not completely identical expression domains [24].

Gene duplications offer several possible outcomes [25]. One option is that one of the copies gets silenced or lost again during evolution. A second option is that one copy retains the ancestral function, freeing the other copy to diverge and evolve new functions (neofunctionalization). A third possibility is that each of the two copies performs a different subset of the ancestral functions (subfunctionalization). The differences in the intrasegmental expression patterns of the two copies in *Cupiennius *suggest that each of the two copies performs different functions that must be the result of either a neofunctionalization or a subfuntionalization event. As there is no genomic information available yet, it remains unclear whether there are additional duplicated Hox genes in *Cupiennius *(see also next section).

An important question is why duplicated Hox genes are present in the spider and why they are retained? Are they remnants of a large duplication event that are maintained because of neofunctionalization or subfunctionalization events? Or is there another evolutionary advantage for the spider to have multiple copies of some Hox genes? Presently it is difficult to answer these questions. In chelicerates there seems to be a tendency towards having more Hox genes [this paper, [13-15]], this in contrast to insects where there is a reduction of true Hox genes as two Hox genes -*Hox3 *and *ftz*- lost their homeotic function and obtained new functions in the insect embryo, which is associated with a divergence of the sequence of the gene [26-28].

Hox gene duplications have been proposed to be one of the genetic mechanisms behind the diversification of vertebrates [e.g. [29]]. However it remains difficult to draw a direct link between Hox gene duplications and morphological evolution. Recent results from Lynch et al [30] suggest an important role for the action of positive Darwinian selection in the divergence of vertebrate Hox genes after cluster duplications. The locations in the homeodomain of the sites that are under positive selection suggest that they are involved in protein-protein interactions. This suggests that adaptive evolution actively contributed to Hox gene function [30]. Indeed, in the *Cs*-Ubx-2 homeodomain there are two amino acid exchanges compared to the homeodomain of *Cs*-Ubx-1 or of Ubx of most other arthropods (Fig 1). Only in the honey-bee and the crustaceans *Moina *and *Artemia *there is one amino acid exchange in the homeodomain, in all three cases an A to S exchange at position 37 of the homeodomain (not shown). Also one of the two exchanges in *Cs*-Ubx-2 is an A to S on position 37. The sequence divergence in the homeodomain of *Cs*-Ubx-2 thus might be associated with a functional divergence. However the mechanism of the divergence is unknown, leaving open the role of Hox gene duplication in morphological evolution of chelicerates.

### Duplicated genes in the spider: a whole genome duplication?

The most important question that comes up now is on the origin of these three duplicated Hox genes in *Cupiennius*. There are two options. First, they result from a duplication of the complete cluster. This would imply that either additional Hox genes are present as two copies that have not be found so far, or that one copy has been lost for the other Hox genes, as has happened to some of the Hox genes in the duplicated vertebrate clusters. Mammals for instance possess four Hox clusters, but most of the paralogs are not present as four copies as some of them have been lost in some of the clusters [11]. All data for *Cupiennius *Hox genes were obtained via either PCR approaches or cDNA library screening [15]. As there is no genome project for the spider yet, this means that it is presently unclear whether additional Hox genes are present as duplicated copies in the spider. The second possible explanation for the three duplicated *Cupiennius *Hox genes could be three independent tandem duplications of the individual genes. Additional analyses are required to identify the genomic organization of the spider Hox genes, and to find out whether these genes are indeed organized in two clusters, or whether the duplicated genes are serial duplications within a single Hox cluster.

However, there is some additional data that point to large-scale duplication of chromosomal fragments or even complete genomes in the spider. So far we also have found in our PCR screens several other genes that are present in two or more copies in the transcriptome of the spider *Cupiennius*, like *extradenticle, homothorax, H15, Wnt5, Wnt7, engrailed, Delta, Suppressor of hairless, Krüppel, runt, pairberry, optomotor blind odd-skipped, apterous, orthodenticle *[[19,31-37], our unpublished data]. In contrast, in most other arthropods most of these genes are present as one copy only. The relative high number of duplicated genes may point to a major duplication event in lineage to the spider, which might be caused by a whole genome duplication. A spider genome project would help to verify this.

### Two posterior expression boundaries of spider Hox genes

Now data from all ten different arthropod Hox gene classes are known from this spider, another fact becomes obvious, that we already recognized previously based on a smaller data set [38], but which becomes even more prominent by new data on *Cs-pb*, *Cs-Scr*, and *Cs-ftz *[this paper, [19]]. There are two discrete posterior expression boundaries for Hox genes in the *Cupiennius *(Fig 6). The expression of all anterior Hox genes (*lab, pb, Hox3, Dfd, Scr, ftz*) ends at the boundary between fourth walking leg (L4) and first opisthosomal segment (O1), which is at the tagma boundary between prosoma and opisthosoma. Also the posterior Hox genes (*Antp, Ubx, abdA, AbdB*) all have the same posterior expression border: the very posterior end of the embryo. There is only one Hox gene, *Cs-Antp*, that crosses the tagma boundary (Fig 6). In other arthropods, but also in vertebrates, most of these posterior expression borders are not defined as well as in the spider [12,39].

The reason for the two discrete posterior expression borders remains unclear and we only can speculate on this. Between L4 and O1 is an important morphological boundary, the one between the two tagmata of the spider: the prosoma and the opisthosoma. The Hox genes might play a role in the specification of this boundary. In contrast, several Hox genes cross tagmata borders in other arthropods [e.g. [12,23]]. If the Hox genes play a role in tagma border specification, then this must be a peculiarity of the spider.

Another explanation could be that the anterior Hox genes are required for the specification of the different appendages in the spider. All six anterior Hox genes are expressed in distinct patterns within the appendages suggesting a role of them in appendage specification [[15,17,19], this paper] (see also Fig 3 and 4). It has been shown that Hox gene expression is associated with morphological diversification of leg segments in insects [40]. Indications for interactions between Hox genes in the spider legs come also from the weaker expression of *Cs-Dfd-2 *in L3 and L4 that coincides with the stronger expression of *Cs-Scr-1*, *Cs-Scr-2 *and *Cs-ftz *in L3 and L4 (Fig. 3F, Fig. 4C, Fig. 4F and reference [19]. Thus there might be a cross regulation between these Hox genes in the legs. Such a role of these Hox genes in the legs may form the reason for shared posterior expression boundaries (Fig. 6). The border between segments with and without appendages coincides with the tagma boundary between prosoma and opisthosoma. Spiders have true appendages on six segments: the cheliceres, the pedipalps, and four pair of walking legs. The more posterior segments do not have true appendages, however the second to fifth opisthosomal segment develop limb buds that give rise to the respiratory organs and the spinnerets [41].

A third possible explanation could be that the discrete Hox gene expression boundary is a result of the segmentation process that acts more upstream in the regulatory cascade and that lays down the segments. It is known from insects that the segmentation gene cascade indeed also controls the expression of Hox genes [42-44]. In insects this is mainly done by orthologs of gap genes. It is not known yet what genes regulate the expression of the spider Hox genes. A number of spider Hox genes obey parasegmental boundaries, as they do in *Drosophila *[31]. Parasegmental boundaries are important developmental boundaries in the early embryo and are specified by the segmentation gene cascade [45]. Segment-polarity genes like *wingless*, *cubitus interruptus *and *engrailed *maintain the parasegmental boundaries in arthropods. We assume that at least in part the same upstream acting regulatory machinery controls the segment-polarity genes and Hox genes in the spider as their expression boundaries match exactly. The discrete posterior expression border of the spider Hox genes is the result of genes that control them and these therefore may be an output of the upstream segmentation machinery that also control the expression boundaries of the segment-polarity genes. The assumption that the discrete Hox gene expression boundary could be the result of the segmentation process is strengthened by previous work in *Cupiennius *that suggested that there may be at least partially a difference in the mechanisms that specify the anterior segments and the posterior segments [34]. The posterior segments form sequentially from a posterior growth zone and may be partially regulated in a different way. The discrete boundary of Hox gene expression at the prosoma-opisthosoma boundary therefore could reflect such a difference in the regulation of segmentation between the anterior and the posterior segments.

## Methods

### Spiders and embryos

Embryos were obtained from our *Cupiennius salei *Keyserling (Chelicerata, Arachnida, Araneae, Ctenidae) colony in Cologne. Cocoons with embryos were taken from fertilized adult female spiders, RNA was isolated and reverse transcribed into cDNA as described previously [31], embryos were treated and fixed as described previously [17,31].

### Isolation of additional *Cupiennius salei *Hox genes

Initial PCR fragments of the *Cs-pb*, *Cs-Dfd-2*, *Cs-Scr-1*, and *Cs-Scr-2 *were obtained using primer combinations directed against sequences in the homeodomain. We used the following primer combinations: for *Cs-pb *and *Cs-Dfd-2 *the primers 1521 (5'-GGA TTC TAY CCI TGG ATG-3') and 1520 (5'-CAT ICK ICK RTT YTG RAA CCA-3'), for *Cs-Scr *the primers 1289 (5'-CCN CAR ATH TAY CCN TGG ATG-3') and 1290 (5'-TT CCA YTT CAT NCG NCK RTT WTG- 3'). Additional sequences have been obtained by subsequent RACE-PCR. Additional sequence of the *At-Dfd-1 *gene has also been obtained via RACE-PCR, using primers based on a short sequence published by Abzhanov et al [14]. The clones have been sequenced in both directions and the sequences are available under the accession numbers AM419029 to AM419032.

### Expression analysis

The expression patterns of the genes have been analyzed by in situ hybridizations using digoxigenin labelled anti-sense RNA probes [17]. We used the following probes: for *Cs-Dfd-1*, *Cs-Ubx-1 *and *Cs-Ubx-2 *we used probes prepared from the cDNA clones isolated from the cDNA library [15] (accession numbers CAA07498, CAA07500, CAA07501), for *Cs-pb*, *Cs-Scr-1 *and *Cs-Scr-2 *we used probes prepared from the all available cDNA sequence (accession numbers AM419029, AM419030, AM419031), for *Cs-Dfd-2 *we used a probe prepared from a 3'RACE fragment, corresponding to nt 84–1407 of the available cDNA information (accession number AM419032).

## Competing interests

The author(s) declare that they have no competing interests.

## Authors' contributions

EES and MS carried out the cloning of the *Cs *genes and analysis of the expression patterns. MP recovered the complete *At-Dfd-1 *sequence and helped with the sequence analysis. WGMD conceived of the study, participated in its design and coordination, and wrote the manuscript. All authors read and approved the final manuscript.
